# Evaluation of the effect of particulate matter on construction accidents using relative probability

**DOI:** 10.1038/s41598-023-45358-y

**Published:** 2023-10-23

**Authors:** Minsu Lee, Jaewook Jeong, Daeho Kim

**Affiliations:** 1 Department of Safety Engineering, Seoul National University of Science and Technology, Seoul, 01811 Republic of Korea; 2 Department of Civil and Mineral Engineering, University of Toronto, 35 St. George St, Toronto, ON M5S 1A4 Canada

**Keywords:** Environmental social sciences, Engineering

## Abstract

PM_10_ is known to have a great adverse effect on the human body. However, there is a lack of research analyzing the impact of PM_10_ on the occurrence of accidents. Accordingly, the purpose of this study is to analyze the correlation between PM_10_ and accidents in the construction industry and to present a new concentration group to manage accidents caused by PM_10_ in the construction industry. This study was conducted in the following four stages. (i) collection of data, (ii) classification of data, (iii) relative probability analysis, and (iv) modified PM_10_ group classification. The main results of this study are as follows. When the frequency analysis of the traditional method was conducted, 3,721 accidents occurred at a PM_10_ concentration of 32 μg/m^3^. However, as a result of the relative probability analysis presented in this study, it was confirmed that the relative accident probability increased as the PM_10_ concentration increased. In addition, the current PM_10_ concentration is presented by the WHO in six groups from a health perspective. However, in this study, the newly proposed PM_10_ group was classified into three groups from the perspective of the probability of construction accidents. The group proposed in this study is not from a health perspective but a group for managing construction site accidents. The contribution of this study was to confirm that PM_10_ also affects accidents occurring at construction sites, and the impact of PM_10_ on accidents was quantitatively analyzed through the relative probability analysis presented in this study.

## Introduction

Unlike the manufacturing industry, the construction industry does not have a fixed workplace, and most of the work is carried out outdoors. Therefore, construction workers are exposed to various outdoor environments^1^. These environmental factors have a lot of influence not only on the health of workers but also on labor and productivity^2–6^. Currently, the world recognizes the seriousness of air pollutants effects on the human body and implements policies for regulation and management to manage them. According to the Organization for Economic Cooperation and Development, the number of deaths from air pollutants worldwide was estimated at 3 million in 2010. Awareness of air pollutants is increasing worldwide, with the number expected to increase to 6 to 9 million by 2060^7^. Among them, particulate matter less than 10 microns (PM_10_) has very small particles, so it penetrates deeper into the human body and causes various diseases. As a result, research results that have a serious adverse effect on the human body, such as an increase in mortality, cardiovascular disease, respiratory disease, and cancer incidence, have been reported. Due to this risk, the International Agency for Research on Cancer under the World Health Organization designated and managed PM_10_ as a group 1 carcinogen in 2013^8^. According to the Korea Disease Control and Prevention Agency, for every 10 μg/m^3^ increase in PM_10_ concentration, the hospitalization rate due to Chronic Obstructive Pulmonary Disease increases by 2.7% and the mortality rate by 1.1%. In addition, it is reported that the incidence of lung cancer increases by about 9% when the concentration of particulate matter less than 2.5 microns (PM_2.5_) increases by 10 μg/m^3^, so it is recommended that underlying patients not be exposed for a long time when PM_10_ is severe^9,10^. As such, studies have shown that PM_10_ has a serious impact on the human body, which also adversely affects workers' productivity. When air pollutants increase by 1% at production facilities in China, productivity decreases by about 0.1%^11^.

According to past literature on fine dust and productivity, air pollution, especially PM_10_, was found to have a significant impact on labor productivity in a study that analyzed the correlation between air pollution, socioeconomic factors, and labor insurance and labor productivity in Taiwan^12^. In addition, it was found that every 10 μg/m^3^ increase in fine dust resulted in 20 deaths and about $600,000 in damage^6^.

Past literature analyzing the effects of PM_10_ on the human body has shown that PM_10_ has a significant impact on deaths from asthma and cerebrovascular diseases. Next, studies have shown that PM_10_ has a significant impact on mortality, early death, and hospital admissions^13–19^.

As such, if PM_10_ has a large impact on the human body and affects productivity, research is needed on whether it also affects workers' accidents. Most of the previous studies related to PM_10_ conducted in the past were health-related and economy-related studies, such as the effect of PM_10_ on health^20–29^, labor productivity^2–6^, and economic impact^12,30^. If PM_10_ affects workers in various ways, it is necessary to analyze and manage whether it affects the occurrence of accidents. However, there are currently no research cases that have evaluated the quantitative impact of PM_10_ on the occurrence of accidents.

Most of the past research topics related to PM_10_ have been health issues, such as health and productivity. If it can affect productivity, it can also affect the occurrence of accidents, but there are no research cases on this. However, there are currently no studies analyzing the effect of PM_10_ on the occurrence of accidents.

In the past, the higher the concentration of PM_10_ and NO_2_, the more accidents occurred, but it was presented only in terms of frequency, not in terms of probability^31^.

Therefore, the purpose of this study is to analyze the correlation between PM_10_ concentration and accidents occurring at construction sites in terms of frequency and probability. In addition, the PM_10_ concentration group is currently managed based on its impact on the human body. In this study, a new PM_10_ concentration group is presented based on construction accidents caused by PM_10_ at construction sites.

## Materials and methods

This study was conducted in the following four steps: (i) collection of data, (ii) classification of data, (iii) relative probability analysis, and (iv) modified PM_10_ group classification.

### Collection and methods

The data collected included injuries, accidents, and PM_10_ concentrations.

Injury and fatality data were collected based on the industrial accident compensation data of the Korea Occupational Safety and Health Agency. At this time, accidents caused by diseases were excluded, and accidents were analyzed. Data were collected on 6736 fatalities and 207,802 injury accidents from 2007 to 2019, for a total of 214,538 accident records^32^.

After that, the Korea Meteorological Administration collected PM_10_ concentrations for all days from 2007 to 2019. After that, the PM_10_ concentration on the day of the accident was matched. In Korea, there are monitoring stations that measure the concentration of PM_10_ on a city-level basis in all regions. Therefore, we were able to find the relative probability of accidents according to the concentration of PM_10_ for all days and all regions^33^.

The reason for the recent failure to use data is that the total number of accidents at construction sites in Korea has been announced, but no detailed statistics have been released on what date and time the accident occurred in which area. Therefore, it did not reflect the latest data. However, I had the opportunity to collect accident data prior to 2019, so I used the data for that period. This study analyzes the effect of fine dust on accidents. Therefore, the number of data points is considered more important than the latest data. In this study, more than 200,000 data points were collected, so enough data was collected to correlate. Future studies will try to collect recent data.

### Classification of data

Currently, PM_10_ is managed based on its impact on the human body. The World Health Organization (WHO) is managing it in six stages in terms of health. Table 1 below is a table that presents the concentration group for WHO to manage PM_10_ in terms of health^34^. The PM_10_ concentration collected over 13 years was 1 μg/m^3^ to 123 μg/m^3^, excluding missing data. As a result of the classification of the PM_10_ concentrations collected in this study, they were classified into five groups according to the WHO standard, ranging from "good" to "very unhealthy." In this study, the relative accident probability for each concentration is analyzed. Therefore, the number of accidents and the relative probability were analyzed in units of 1 μg/m^3^.

**Table 1:** PM_10_ concentration group presented by WHO.

Concentration (μg/m^3^)	Under 30	30 to 49	50 to 89	90 to 119	120 to 154	Over 155
Grade	Good	Moderate	Unhealthy for sensitive people	Unhealthy	Very unhealthy	Hazardous

The classification system presented by the WHO as above is a concentration group proposed for PM_10_ management in terms of health. Through this concentration group classification system, the health management of workers at construction sites is carried out. However, assuming that it affects accidents occurring at PM_10_ construction sites, it cannot be applied as a safety management indicator to manage accidents at construction sites through this concentration group. Therefore, this study quantitatively analyzes the effect of PM_10_ on the occurrence of accidents during construction projects and newly classifies PM_10_ concentration groups for safety management in terms of accidents.

### Relative probability analysis

This study aims to analyze the correlation between PM_10_, which was previously managed and studied in terms of health, and accidents occurring at construction sites. To this end, a relative probability analysis was conducted. Existing frequency analysis can analyze how many accidents have occurred for each concentration of PM_10_, but it is not suitable for analyzing the probability of accidents. When assessing risk, consider probabilistic aspects as well as frequency^35^. Therefore, in this study, a relative probability analysis was introduced and conducted to quantitatively analyze the effect of PM_10_ concentration on accidents occurring at construction sites. The proposed relative probability analysis is calculated through Eqs. (1) through (3) below^36,37^. Through this, it is possible to quantitatively evaluate the relative probability of accidents occurring at construction sites by PM_10_ concentration.

1 $$Accident ~rate=\frac{Number ~of ~accidents~ by ~air~ pollutants}{Total~ number~ of ~accidents}$$

2 $$Period ~rate=\frac{Number ~of ~times ~by~air~ pollutants}{Total ~number~ of~ times ~in ~13~ years}$$

3 $$Relative ~probability=\frac{Accident~ rate}{Period~ rate}$$

Here, the incident ratio was calculated by using the total number of accidents as a denominator and the numerator as the number of accidents by fine dust concentration. Next, the Period ratio was calculated using the total number of days for the entire period, that is, 13 years as a denominator and the number of days of appearance by fine dust concentration as a numerator. Finally, Relative probability was calculated using the Period rate as a denominator and the Accident rate as a numerator.

By calculating the relative probability through the above equation, the baseline can be set to 1. What Baseline 1 means is that the probability of an accident is equal to the overall average probability of an accident. If the relative probability value is higher than 1, it means that the probability of other accidents is relatively higher than the average. Conversely, a reading below 1 means that the probability of an accident is relatively low^36,37^.

### Modified PM_10_ group classification

As mentioned above, the current PM_10_ concentration is classified and managed by the WHO into six groups based on its effect on the human body. In this study, a new concentration group was presented based on the probability of accidents occurring at construction sites, not the effect of PM_10_ on the human body.

The following two steps were performed to present a new concentration group. First, conduct K-means clustering. Second, conduct an ANOVA analysis. A description of each step of the study's progress is as follows.

#### Conducting k-means clustering

In this study, a new concentration group was proposed based on the effect of PM_10_ on the occurrence of accidents at construction sites, and the data were classified into a new group using K-means clustering. The reasons for the new proposal for the PM_10_ concentration group are as follows. Currently, the concentration of PM_10_ is presented by the WHO from the perspective of its impact on the human body. In other words, it is an effective indicator for health management at construction sites, but it is inappropriate as an indicator for managing accidents. If PM_10_ affects accidents at construction sites, indicators that can be managed to prevent accidents need to be presented. Therefore, in this study, we present a new concentration group that can manage the impact of accidents through K-means clustering.

K-means clustering is an algorithm that binds given data into K clusters. It is one of the representative separable clustering algorithms, and each cluster has one centroid. Each object is assigned to the nearest center, and the objects assigned to the same center gather to form a single cluster, at which time the user must directly determine the number of clusters needed to execute the algorithm^38^.

As such, K-means clustering has the limitation that the number of clusters must be set according to the user's experience and subjectivity. The elbow point method and silhouette score allow you to quantitatively set the number of clusters in K-means clustering.

In this study, the elbow point method was used to determine the number of clusters in K-means clustering. Elbow points can then be determined by hierarchical clustering. Hierarchical clustering is used when the number of clusters is not determined, and you can determine the number of clusters based on elbow points. The reason for finding the elbow point is that as the number of clusters increases, the distance sum within the cluster decreases, and it is important to find a point where the distance sum does not decrease significantly even though the number of clusters increases. K-means clustering determines the number of clusters by finding where the sum of squared errors (SSE) is minimized. At this point, hierarchical clustering allows you to determine the point at which the SSE is minimized by checking the elbow points. SSE is calculated using Eq. (4)^39,40^.

4 $$SSE = \mathop \sum \limits_{i = 1}^{n} \mathop \sum \limits_{{x \in c_{i} }} dist(x, c_{i} )^{2} ,$$

where SSE is the sum of squared error, k is the number of clusters, x is the set of observations, and ci is the center of each cluster.

#### Significance verification through Anova analysis

Analysis of Variance (ANOVA) is a statistical analysis methodology used to determine the difference between the means of three or more groups. Before you can perform an ANOVA, you must perform Levene's ANOVA test to determine the equal variance of the data you want to analyze. If the data are not distributed equally, you should use the Welch test instead of an ANOVA to verify the mean difference between each group^40^.

In addition, post-hoc analysis must be performed to determine the difference in mean between groups by performing an ANOVA. If the data is equally distributed during post-hoc analysis, the Scheffe and Duncan tests can verify the mean difference between each group. Games-Howell should be used for post-hoc if Levene's Test for Equity of Varians is used to confirm that it is not an equal variance^41^.

In this study, an ANOVA was conducted to determine whether the newly proposed PM_10_ concentration group was statistically significant using K-means clustering. At this time, the newly proposed PM_10_ concentration group is not the concentration group previously presented based on the impact on the human body but the concentration group presented for accident management at the construction site. As a result of conducting Levene's Test for Equity of Varians on the collected data, it was confirmed that there was no equal variance, so Welch's test was used. Afterward, the difference in mean between groups was confirmed using Games-Howell for follow-up analysis.

## Results

### Frequency analysis based on the concentration of air pollutants

Using the methodology presented in Section "Materials and methods" above, the relative probability was used to analyze the accident data that occurred at the construction site for 13 years. Prior to the relative probability analysis, the number of accidents caused by PM_10_ concentrations was first identified through frequency analysis.

**Figure 1:**
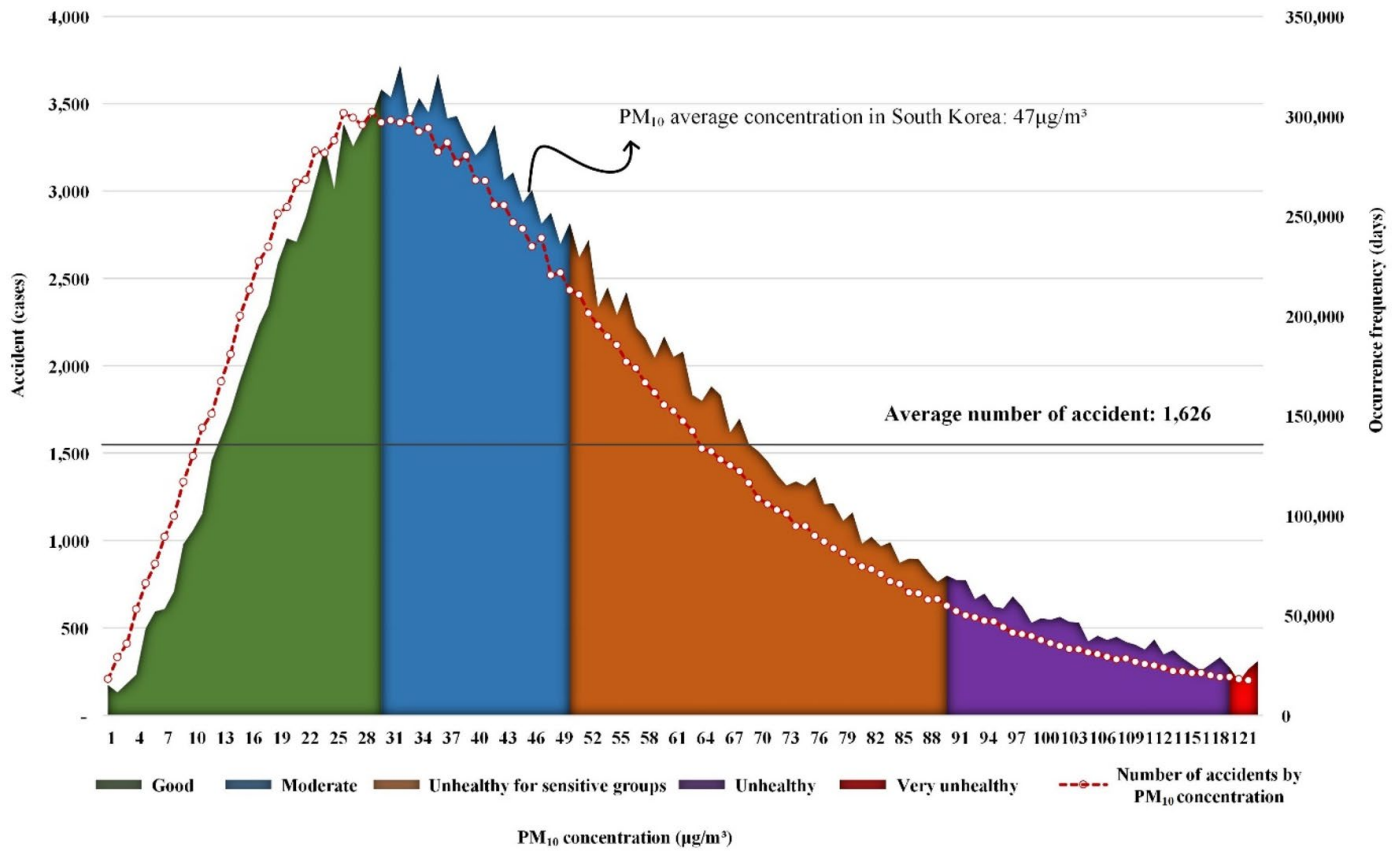
Analysis of accident frequency and appearance frequency by PM_10_ concentration based on WHO.

Figure 1 above shows the results of a frequency analysis to analyze the number of counts by PM_10_ concentration. The x-axis is the concentration of PM_10_, and the y-axis is the number of accidents. The auxiliary axis indicates how much the corresponding PM_10_ concentration appeared over 13 years. The area chart represents the number of accidents by PM_10_ concentration, and the gray horizontal line represents the average number of accidents. Finally, we specified colors for each group managed by WHO: “Green” as "Good”, “Blue” as "Moderate”, “Orange” as "Unhealthy for sensitive groups”, “Purple” as "Unhealthy" and “Red” as "Very unhealthy”.

The results of the accident frequency analysis by PM_10_ concentration are as follows.

The accident occurred in the "good" to "very unhealthy" group, and it was confirmed that no accident occurred in the "hazardous" group. The concentration that caused the most accidents was 32 μg/m^3^, with 3,721 cases. The average concentration of PM_10_ in Korea is 47 μg/m^3^, which is higher than 32 μg/m^3^, which is the concentration at which the most accidents occurred^42^.

### Calculation of relative probability by air pollutants concentration

It is difficult to quantitatively determine the effect of the PM_10_ concentration on the accident only by analyzing the frequency of accidents by PM_10_ concentration. Therefore, it is necessary to quantitatively analyze the probability of accidents by PM_10_ concentration in consideration of the same period. In this section, we present the results of calculating the relative probability of PM_10_ contributing to the accident through the relative probability analysis using the methodology presented in Section "Relative probability analysis".

**Figure 2:**
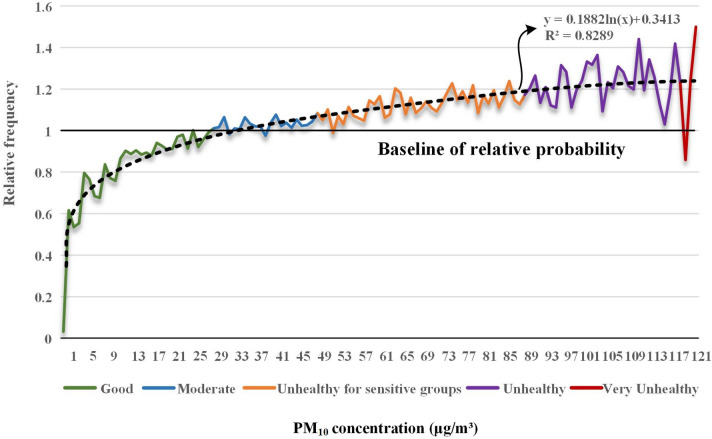
Relative probability analysis results according to PM_10_ concentration.

Figure 2 is a graph that analyzes the relative probability by PM_10_ concentration. The x-axis represents the concentration of PM_10_, and the y-axis represents the relative probability. The line graph represents the relative probability of the corresponding PM_10_ concentration. Each color is a PM_10_ concentration group suggested by the WHO based on its effect on the human body: “Green” as “Good,” “Blue” as “Moderate,” “Orange” as “Unhealthy for sensitive groups,” “Purple” as “Unhealthy,” and “Red” as “Very unhealthy” group. A black horizontal line indicates baseline 1, which is the baseline probability of accident occurrence for air pollutants. The black-dotted line represents the trend line.

The results of the relative probability analysis to quantitatively evaluate the probability of accidents caused by PM_10_ at construction sites are as follows.

According to the PM_10_ frequency analysis results presented above, 3,721 cases occurred at 32 μg/m^3^, which is the concentration with the most accidents. However, in the analysis of the PM_10_ relative probability, it was confirmed that the relative probability also increased as the concentration increased. The PM_10_ concentration with the highest relative probability value was 1.50 at 123 μg/m^3^. This means that during the same period, the incident was 1.50 times higher than the average (baseline 1) at that concentration. As a result of a regression analysis on the PM_10_ relative probability, the R^2^ value was 0.83, showing high explanatory power. Therefore, as the PM_10_ concentration increases, the relative probability of an accident increases.

### Determining the number of clusters in air pollutants

Currently, the PM_10_ concentration group presents a rating based on its effect on the human body. This is appropriate as an indicator for the health management of workers at construction sites but inappropriate as an indicator for managing accidents. This study quantitatively analyzed how PM_10_ affects the occurrence of accidents at construction sites. Therefore, in this study, after quantitatively analyzing the effect of PM_10_ on accidents occurring at construction sites, a new PM_10_ concentration group was clustered and presented based on the results.

At this time, two cluster methodologies were developed and analyzed. First, hierarchical cluster analysis was performed to identify the elbow point and derive the K value of the K-means cluster. Second, a new concentrated group was presented by performing a K-means cluster based on the values confirmed using the elbow point.

**Figure 3:**
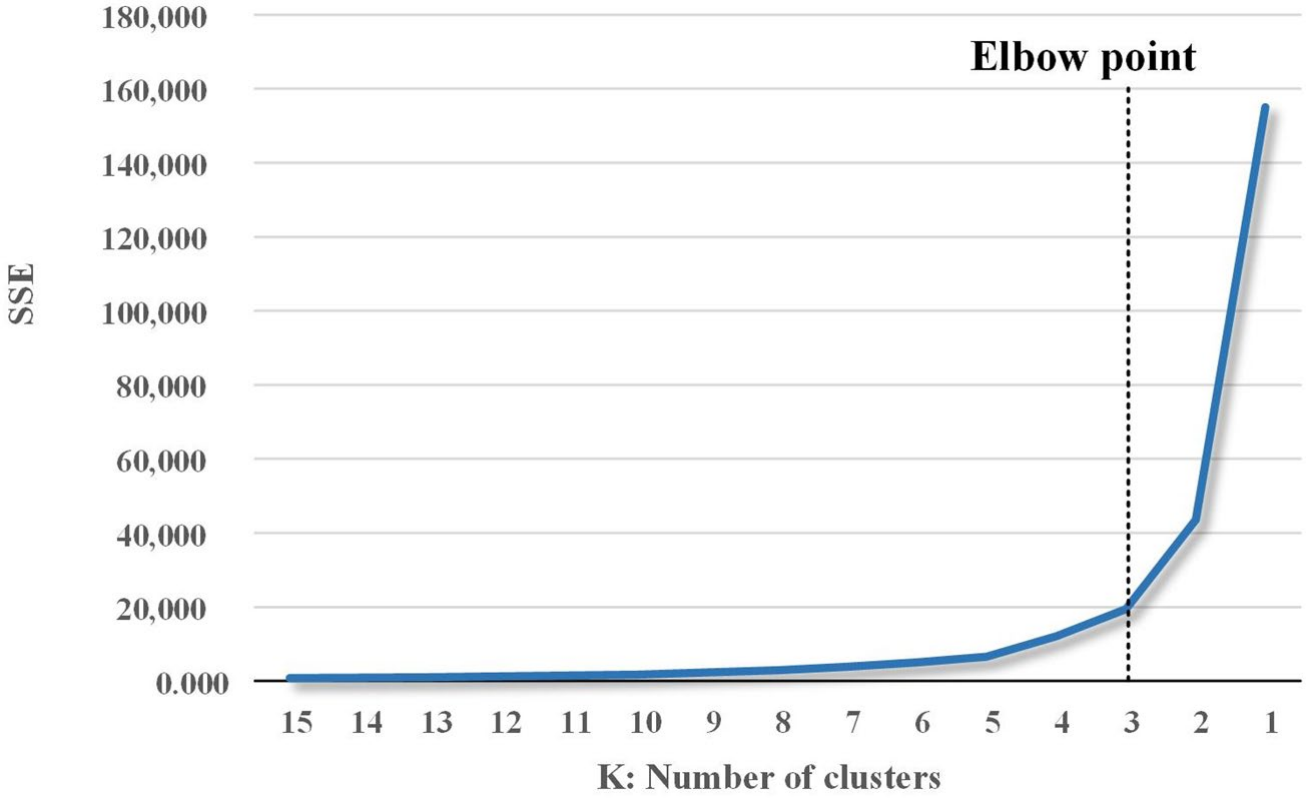
Elbow-point results for new PM_10_ concentration group classification.

Figure 3 shows the elbow-point results derived by performing hierarchical clustering on the collected PM_10_ data. The value of elbow-point is determined by the point at which the SSE decreases rapidly before stabilization^12^. The elbow-point result value of PM_10_ was derived as 3, and the number of clusters in the K-means clustering was determined to be 3 groups.

**Figure 4:**
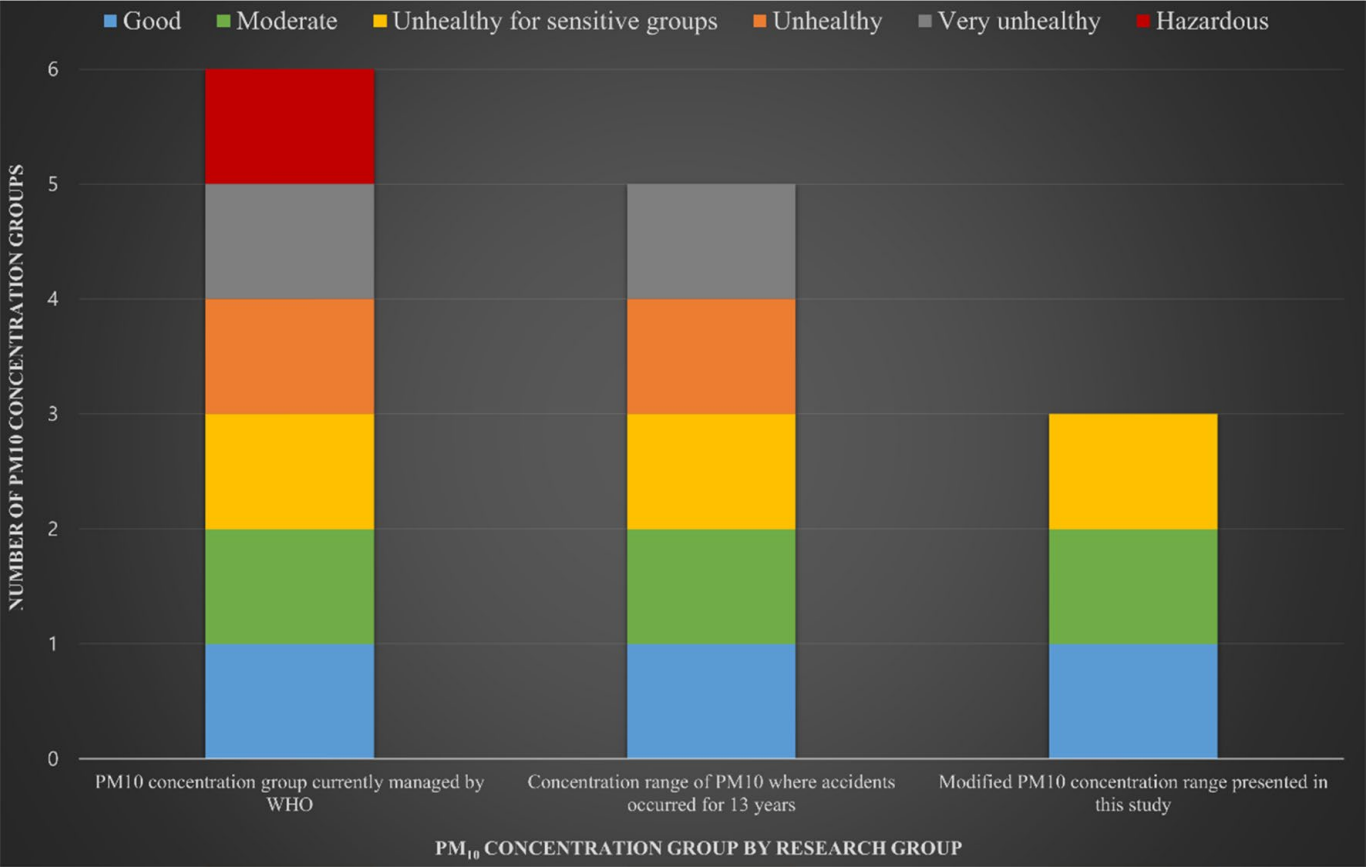
Differences in PM_10_ concentration groups by study procedure.

Figure 4 shows the PM_10_ concentration group presented as this study progresses. From the left, (1) the PM_10_ concentration group currently presented by the WHO, (2) the PM_10_ concentration group collected based on the accident data collected in this study, and (3) the PM_10_ concentration group newly proposed in this study.

Currently, the WHO manages and presents the effect on the human body, that is, the PM_10_ concentration group, from a health perspective. This is a suitable indicator in terms of health management at construction sites. However, according to the results of Section "Calculation of relative probability by air pollutants concentration" above, it was confirmed that the relative probability of an accident increases as the PM_10_ increases. Therefore, not only indicators for health management at construction sites but also indicators for managing accidents are needed. Therefore, it can be interpreted that the concentration group proposed by the WHO is not accurate in managing accidents caused by PM_10_ at the current construction site. In this study, we presented indicators for managing accidents caused by PM_10_ at construction sites. As a result, there are currently six concentration groups proposed by the WHO, but five PM_10_ concentration groups were collected in this study, and three PM_10_ concentration groups were newly proposed in this study.

### Proposal of a new group according to the effects of air pollutants on accidents in the constructure industry

**Figure 5:**
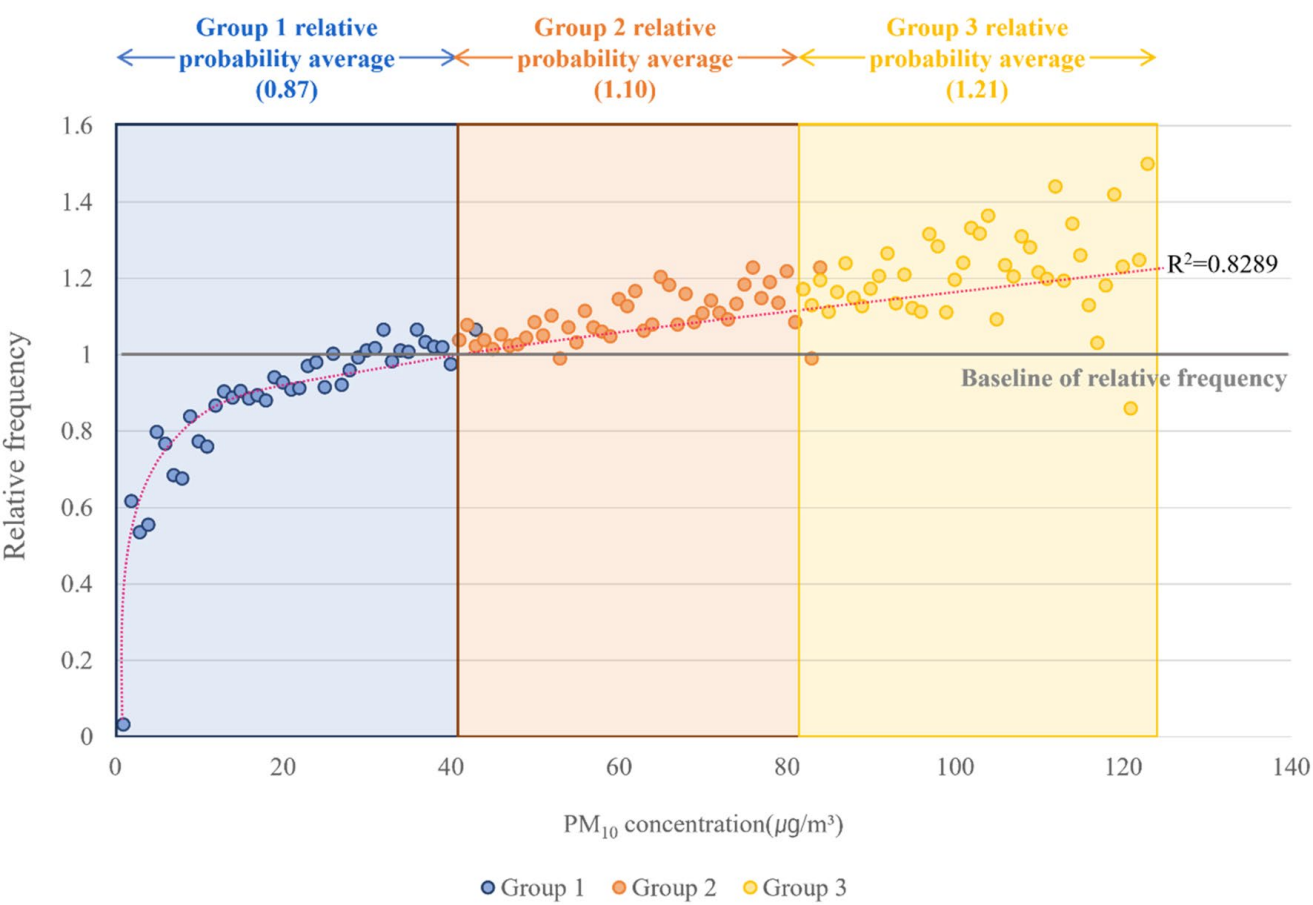
The average relative probability of PM_10_ groups and groups newly proposed in this study.

In order to confirm the newly presented PM_10_ group, K-means clustering was performed using the number of clusters identified in Section "Determining the number of clusters in air pollutants". In 3.3, the number of clusters was confirmed, and Fig. 5 below presents the range of each concentration group based on the concentration of PM_10_ at which accidents occurred in the past. The proposed concentration group is the newly proposed PM_10_ concentration group based on the accident at the construction site.

In the graph, the x-axis represents the PM_10_ concentration, and the y-axis represents the relative probability. The line graph shows the relative probability of PM_10_ concentration. Blue is modified group 1, orange is modified group 2, and yellow is modified group 3. The black horizontal line indicates a baseline of relative probability.

Based on the relative probability of PM_10_, the newly proposed concentration groups were identified as three, less than the six previously suggested by the WHO. For each group, the concentration range is 1 μg/m^3^ to 40 μg/m^3^ for the first group, 41 μg/m^3^ to 81 μg/m^3^ for the second group, and 82 μg/m^3^ to 123 μg/m^3^ for the third group. If the relative probability of an accident is calculated using the relative probability presented in this study, a baseline is presented. The reference line is 1, and if the relative probability is less than 1, it means that the accident probability is relatively low. Conversely, a value higher than 1 means that the probability of an accident is relatively high, which means that it is a concentration that requires management. As a result of relative probability analysis, it was confirmed that, unlike frequency analysis, the higher the concentration of PM_10_, the higher the probability of accidents.

The average values of the relative probabilities for each group were 0.87, 1.10, and 1.21. This means that the accident probability of PM_10_ concentration in the first group is lower than the average during the same period, and the accident probability of PM_10_ concentration in the second and third groups is higher than the average accident probability.

### Statistical significance verification through ANOVA analysis

In this section, an ANOVA was conducted to determine whether the newly proposed PM_10_ concentration group was statistically significant.

Tables 2 and 3 show the results of ANOVA and post-hoc tests to determine whether there is a statistically significant difference in the modified PM_10_ group. As a result, as shown in Table 2, the difference in means between the three groups was confirmed. Next, as shown in Table 3, post-hoc analysis was conducted using Games-Howell. As a result, the difference in mean between the three groups was confirmed. This result means that the newly proposed modified PM_10_ group is effective when evaluating the probability of an accident by considering the PM_10_ concentration at the construction site.

**Table 2:** ANOVA Results for Modified PM_10_ Concentration Group.

Dependent variable	Group	Number of data	Average	Levene’s test	Levene’s *p*	Welch’s test	Welch’s* p*
PM_10_	1	41	0.87	7.14	.001	49.21	.000
	2	42	1.10				
	3	43	1.21

**Table 3:** Post-hoc analysis (Games Howell) Results of Modified PM_10_ Concentration Group.

Modified PM_10_ group	1	2	3
1	–	.000	.000
2		–	.000
3			–

## Discussion

This study collected construction site accident data from 2007 to 2019 to analyze the impact of PM_10_ on construction site accidents. At this time, accident data due to disease was excluded from the correlation analysis between disaster accidents and PM_10_. Next, PM_10_ concentrations for all days were collected during the same period. At this time, the PM_2.5_ concentration has been provided by the Korea Meteorological Administration since 2015 and has been provided to all regions since 2018^42^, so it was excluded from this study. The exclusion is because the collected accident and disaster data and the PM_10_ concentration period are different. Data collected in this study were collected from the Korea Occupational Safety and Health Agency and the Korea Meteorological Agency, which are public institutions in Korea. The Korea Occupational Safety and Health Agency is an institution that manages industrial accident statistics in Korea, and the Korea Meteorological Administration collects and provides same-day figures for weather and air pollutants in Korea. The Korea Meteorological Administration also provides historical data, so the concentration of PM_10_ on the day of the accident was collected and analyzed^43^.

According to the frequency analysis results presented in Section "Frequency analysis based on the concentration of air pollutants", the most accidents occurred near the average concentration in Korea. It was also confirmed that the number of days of appearance and the number of accidents almost matched. This means that frequency analysis alone makes it difficult to quantitatively determine the effect of concentration on the occurrence of an accident. This is because the number of days corresponding to the concentration has been high, so the number of accidents is also high. Therefore, in this study, the concept of relative probability was introduced to quantitatively evaluate the accident probability based on PM_10_ concentration.

As a result, it was confirmed that the higher the PM_10_ concentration, the higher the probability of accidents. As a result of regression analysis, the R^2^ value was 0.8289, which was higher than 0.8, confirming high explanatory power. As a result of checking the newly modified PM_10_ concentration group, it was classified into three groups, less than the six previously proposed by the WHO. This indicates that the PM_10_ concentration group previously managed by the WHO is not appropriate to manage accidents at construction sites. Subsequently, ANOVA and post-hoc tests were performed to confirm statistical significance. As a result, statistical significance was confirmed, which means that the newly proposed PM_10_ concentration group can be used as a new indicator of accident management at construction sites. As a result, statistical significance was confirmed, which means that the newly proposed PM_10_ concentration group can be used as a new indicator of accident management at construction sites. This result means that PM_10_ affects accidents as well as health aspects. It also suggests the need for construction site management.

Past literature has shown that the risk of traffic accidents increases by up to 35% as PM_10_ and PM_2.5_ levels increase^44^. In addition, studies showed that the risk of accidents for outdoor workers increased by 2.5% and 6.4%, respectively, with an increase in PM_10_ and NO_2_. However, there was a limitation in that all past literature did not provide accurate thinking factors^45^. There was an opinion that accidents would increase due to the influence of the human body caused by PM_10_.

This study also quantitatively suggested that PM_10_ affects accidents at construction sites. However, there is a limitation in that it has not been able to analyze the increase in the probability of accidents due to certain factors of PM_10_. It can be assumed that it is due to the lack of visibility due to PM_10_, the decrease in attention, and the effect on physiological and psychological cognitive functions. Future studies will analyze the factors of PM_10_ that affect accidents at construction sites.

Currently, the Ministry of Employment and Labor of the Republic of Korea is protecting the health of outdoor workers by presenting standards according to PM_10_ concentrations. As such, the risks caused by PM_10_ are widely known to the public and the private sector, and management and regulation are being conducted to prevent them. However, there is currently no paper that quantitatively presents the effect of PM_10_ on accidents. Therefore, research is needed to present these criteria and risks^45^.

## Conclusion

This study quantitatively analyzed how PM_10_ affects accidents at construction sites. In addition to traditional frequency analysis, we introduce the concept of relative probability to consider probability aspects. Currently, WHO's PM_10_ concentration group is managed and presented in terms of health. However, in this study, a new concentration group was proposed based on the PM_10_ accident at the construction site.

This study was conducted in the following four steps. (1) collection of data, (2) classification of data, (3) relative probability analysis, and (4) modified PM_10_ group classification.

The results of this study are as follows:

- As a result of conducting the existing frequency analysis of PM_10_, 3,721 accidents occurred with 32 μg/m^3^. However, a relative probability analysis showed that as the concentration increased, so did the relative probability. At this time, the concentration with the highest relative probability is 123 μg/m^3^, which is the highest concentration collected in this study. In addition, regression analysis was conducted, and the R^2^ value showed a high explanatory power of 0.8 or higher.
- As a result of presenting a new concentration group of PM_10_, the significance was verified by checking the average difference between groups by dividing them into three groups and performing a variance analysis. This means that the concentration group presented in this study is suitable for use in accident management by PM_10_ at construction sites.

The contribution point of this study was to confirm the relationship between the probability of an accident and PM_10_, which is currently managed from a health perspective. This is a new research topic that was not discussed in previous studies, and a new indicator was presented to prevent construction worker accidents caused by PM_10_. In addition, the relationship between PM_10_ concentration and construction accidents was quantitatively analyzed based on actual data, and based on this, PM_10_ can be effectively managed at construction sites and can help establish safety measures.

The limitation of this study was to quantitatively analyze the correlation between PM_10_ and construction site accidents, but not how PM_10_ affects accidents. For example, this study did not present an analysis of how PM_10_ affects accidents, such as blurred vision due to PM_10_ or whether accidents have occurred due to poor conditions.

Future research directions will present an integrated risk assessment model that considers not only PM_10_ but also other air pollutants. Specifically, air pollutants will be considered for PM_10_, O_3_, NO_2_, CO, and SO_2_. By calculating the relative probability of each air pollutant, the correlation and relative probability of the construction site accident will be calculated. Through the analysis, we will present an integrated risk assessment model for substances that affect accidents at construction sites. The importance of variables in the integrated model will be weighted by analyzing which variables have a significant impact through neural network analysis. At this time, the correlation analysis between air pollutants and construction site accidents will be analyzed through gray relational analysis, and the principal component analysis will be used to model.

## Data Availability

The data generated and analyzed during this research are available from the corresponding author upon reasonable request.
